# Endothelial Cell Behavior Is Determined by Receptor Clustering Induced by Thrombospondin-1

**DOI:** 10.3389/fcell.2021.664696

**Published:** 2021-03-29

**Authors:** Verônica Morandi, Jim Petrik, Jack Lawler

**Affiliations:** ^1^Rio de Janeiro State University (UERJ), Rio de Janeiro, Brazil; ^2^University of Guelph, Guelph, ON, Canada; ^3^Beth Israel Deaconess Medical Center, Harvard Medical School, Boston, MA, United States

**Keywords:** angiogenesis, CD36, syndecan, thrombospondin, CD47, integrin, endothelial cell

## Abstract

The thrombospondins (TSPs) are a family of multimeric extracellular matrix proteins that dynamically regulate cellular behavior and response to stimuli. In so doing, the TSPs directly and indirectly affect biological processes such as embryonic development, wound healing, immune response, angiogenesis, and cancer progression. Many of the direct effects of Thrombospondin 1 (TSP-1) result from the engagement of a wide range of cell surface receptors including syndecans, low density lipoprotein receptor-related protein 1 (LRP1), CD36, integrins, and CD47. Different or even opposing outcomes of TSP-1 actions in certain pathologic contexts may occur, depending on the structural/functional domain involved. To expedite response to external stimuli, these receptors, along with vascular endothelial growth factor receptor 2 (VEGFR2) and Src family kinases, are present in specific membrane microdomains, such as lipid rafts or tetraspanin-enriched microdomains. The molecular organization of these membrane microdomains and their constituents is modulated by TSP-1. In this review, we will describe how the presence of TSP-1 at the plasma membrane affects endothelial cell signal transduction and angiogenesis.

## Introduction

Like most biological processes, formation of the vasculature is temporally and spatially regulated by a balance of the signals that are elicited by stimulators and inhibitors. The temporal changes in the levels of these factors in the microenvironment determines endothelial cell behavior. In normal adult tissue, the endothelium is in a quiescent state, but it can respond rapidly to form new capillaries through a process termed angiogenesis. To facilitate a rapid response, the constituents of the pro- and anti-angiogenic pathways are co-localized to specific regions of the plasma membrane, such as lipid rafts and tetraspanin-enriched microdomains (Garcia-Parajo et al., 2014). The recruitment of specific proteins and lipids to these clusters is a critical determinant of their function. This spatial compartmentalization of the pathway components facilitates the efficient regulation of signal transduction that is essential for correct physiological response.

Thrombospondin 1 (TSP-1) is a founding member of the matricellular family of proteins (Adams and Lawler, 2011). These proteins are expressed primarily at the cell surface where they participate in the dynamic changes that cells undergo in response to extracellular stimuli. TSP-1 is involved in cell-to-cell junctions in synapses and immune cell interactions (Soto-Pantoja et al., 2015; Risher et al., 2018). As an example of the simplest form of cell-to-cell interaction, TSP-1 forms a molecular bridge between integrins on apoptotic neutrophils and CD36 on macrophages (Ren and Savill, 1995). TSP-1 is also highly expressed in supramolecular attack particles that are made by cytotoxic T lymphocytes (Balint et al., 2020). A molecular layer of TSP-1 on the attack particles presumably functions to engage the target cell (Balint et al., 2020). TSP-1 also participates in the lateral association of membrane proteins within a single cell, such as endothelial cells where it affects the association of proteins involved in angiogenesis pathways. Taken together, the data indicate that TSP-1 brings membrane proteins together, either on the same cell or adjacent cells, to regulate cellular behavior.

Thrombospondin 1 is a large multimeric extracellular matrix protein that is expressed at sites of normal tissue development, remodeling, and repair (Adams and Lawler, 2011). In cutaneous wounds, TSP-1 is deposited by platelets to accelerate wound closure (Agah et al., 2002). Its coordinated expression with vascular endothelial growth factor (VEGF) is important for normal ovarian follicular development (Michaely et al., 2004). As a physiological activator of latent TGFβ, TSP-1 is also involved in fibrotic and immune response (Murphy-Ullrich and Suto, 2018). Additionally, TSP-1 expression is altered during pathological conditions such as myocardial infarction and cancer (Frangogiannis et al., 2005; Stenina-Adognravi et al., 2018).

Thrombospondin 1 antagonizes angiogenesis through multiple mechanisms, several of which suppress the bioavailability of VEGF. TSP-1 inhibits MMP9 activity and suppresses the release of VEGF from the extracellular matrix (Rodriguez-Manzaneque et al., 2001). TSP-1 also binds VEGF and supports its clearance through an lipoprotein receptor-related protein (LRP)-dependent mechanism (Greenaway et al., 2007). A significant portion of the direct anti-angiogenic activity of TSP-1 maps to the three, thrombospondin type 1 repeats, designated 3TSR (Russell et al., 2015). These repeats, which were first identified in TSP-1, contain binding sites for TGFβ and CD36 (Tan et al., 2002). As described below, the binding of TSP-1 to CD36 activates apoptosis pathways in endothelial cells.

The ability of TSP-1 to strongly bind heparin has long been identified and used as a strategic tool for purifying the protein from human platelets (Lawler et al., 1978; Sipes et al., 2018). Physiologically, this affinity for heparin may reflect the capacity of TSP-1 to bind to heparan sulfate (HS) and to heparan sulfate proteoglycans (HSPG; Sun et al., 1989). Binding to HS/HSPG has direct consequences for the role played by TSP-1 in endothelial cell adhesion, proliferation, motility, differentiation, and in the modulation of activity of angiogenic growth factors (Vischer et al., 1988; Vogel et al., 1993; Nunes et al., 2008).

The globular pentraxin-like heparin-binding N-terminal domain (HBD) of TSP-1 bears the main high-affinity sites for binding to heparin/HS (Clezardin et al., 1997). A prototypical BBXB consensus heparin/HS-binding motif (where B represents basic amino acids) in the HBD is represented by the amino acid sequence MKKTRG (residues 79-84), while the sequences ARKGSGRR (residues 22–29) and TRDLASIARLRIAKGVNDNF (residues 170–189) lack this BBXB configuration. However, both are enriched in positively charged residues, essential for the electrostatic interactions playing a major role in the binding of heparin/HS to proteins (Munoz and Linhardt, 2004). Heparin-binding sites have also been identified in the three type-I repeats or properdin-like domains (Guo et al., 1992; Yu et al., 2000) but, while studies have confirmed the essential requirement for the HBD in the interaction of TSP-1 with heparin/HS chains, it remains unclear whether these secondary heparin binding sites are indeed functional in the intact molecule (Yu et al., 2000). So far, TSP-1 interactions with perlecan (Vischer et al., 1997; Feitsma et al., 2000), decorin (Winnemoller et al., 1992; Merle et al., 1997), and some members of the syndecan (SDC) family of HSPG (see below) have already been described.

**FIGURE 1:**
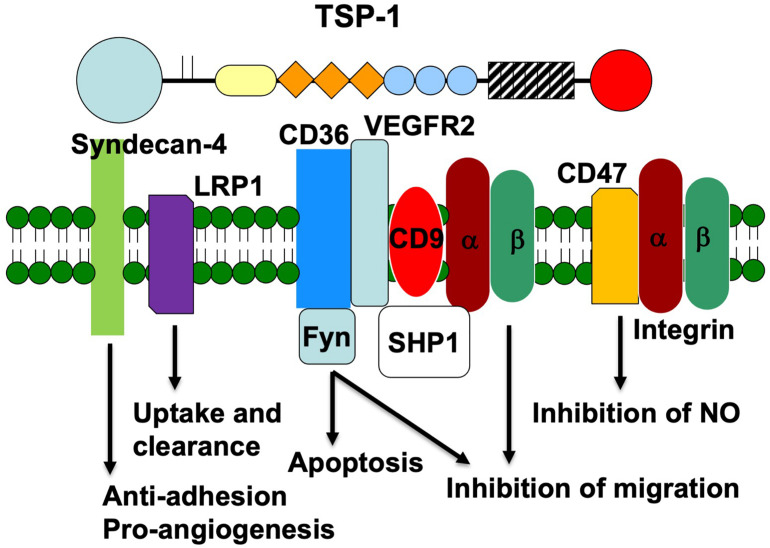
Schematic representation of the endothelial cell receptors that interact with the various domains of TSP-1. The receptors are placed under the domain with which they interact. The signaling proteins that associate with each receptor and the signal that is transduced are also indicated.

In this review, we will focus on the well-characterized interactions of TSP-1 with SDCs, lipoprotein receptor-related protein 1 (LRP1), CD36, integrins, and CD47 in the context of endothelial cell phenotype and angiogenesis (Figure 1). We will discuss how the downstream signals elicited by engagement of these receptors are integrated to determine endothelial cell behavior.

## Syndecans as Receptors for TSP-1

Mammalian SDCs comprise four cell surface members bearing type-I (single-pass) core proteins, with highly conserved short cytoplasmic domains, a transmembrane domain and a varying length extracellular domain, covalently substituted with HS chains (chondroitin or dermatan-sulfate chains may also be present occasionally, to a lesser extent) (Couchman, 2003). SDCs act as receptors or coreceptors for extracellular matrix, growth factors, chemokines, interleukins, and morphogens (Chung et al., 2016) and can engage laterally with other classes of receptors, such as integrins and tyrosine kinase receptors for growth factors (FGFR1, EGFR, among others) (Couchman, 2003), thus contributing to the triggering and modulation of pivotal signaling including PKCα, Akt, and Wnt pathways (Gondelaud and Ricard-Blum, 2019). Indeed, there is growing evidence that SDCs are key parts of processes that involve tissue remodeling, such as development, cancer, inflammation, and tissue repair (Chung et al., 2016). TSP-1 bound to a HSPG recognized by a monoclonal antibody later shown to react specifically with the SDC1 isoform, in mammary epithelial cells (Sun et al., 1989). Both molecules were also found colocalized during lung development (Corless et al., 1992).

Although all four isoforms of SDC have been to some extent implicated in the modulation of endothelial angiogenic differentiation (De Rossi and Whiteford, 2014), a major role for syndecan-4 (SDC-4) seems to prevail over other SDC isoforms, in the context of angiogenesis associated with inflammatory and mechanical stimuli, as well as with injuries occurring in the cardiovascular system (Matsui et al., 2011; Vuong et al., 2015; Russo et al., 2020). SDC-4-null mice exhibit delayed dermal wound repair and defective angiogenesis (Echtermeyer et al., 2001).

Among SDCs, SDC-4 isoform has a unique role in the formation of fibronectin (FN)-induced focal adhesions, in cooperation with β1-integrin receptors (Woods and Couchman, 1994). As other matricellular proteins found in the ECM, TSP-1 destabilizes focal adhesions (Murphy-Ullrich and Sage, 2014), and this activity was specifically located in the HBD domain of TSP-1, in endothelial models (Murphy-Ullrich et al., 1993; Vogel et al., 1993). Interestingly, pro-angiogenic activities of TSP-1 have also been attributed to HBD (Chandrasekaran et al., 2000; Taraboletti et al., 2000; Ferrari do Outeiro-Bernstein et al., 2002).

A 18 kDa HBD comprising the amino acid sequence 1–174—but not intact TSP-1—was able to stimulate tubulogenesis (Ferrari do Outeiro-Bernstein et al., 2002) when physically incorporated into fibrin plugs, a 3D support largely considered as a “provisional matrix” comparable to inflammatory edema and tumor microenvironments (Dvorak, 2015). The two sequences known for both containing affinity for GAGs (TSP-HepI, aa 17–35 and TSP-HepII, aa 78–94) and destabilizing focal adhesions (Murphy-Ullrich et al., 1993) retained the major pro-angiogenic activity of HBD (Nunes et al., 2008). Competitive binding assays indicated that the two TSP-1 motifs could exert their effects by interfering with the recognition of the high-affinity C-terminal heparin-binding domain of FN (FN HepII) by cell surface SDC-4. However, it is important to note that this interference in the action of FN did not affect the maintenance of cell viability, since pathways activated by SDC-4 in its regular role in focal adhesions, e.g., its ability to promote the sequential activation of protein-kinase C-α (PKC-α, a hallmark of SDC-4 activation) and phosphoinositide 3-kinase (PI3K), which in turn activates Akt/protein-kinase B (PKB; Oh et al., 1997; Ilan et al., 1998), were also activated by the HBD domain and its angiogenic peptides TSP-HepI and TSP-HepII (Nunes et al., 2008). These data suggest that, in tissue remodeling microenvironments, the N-terminal HBD possibly generated by proteases may provide the appropriate level of adhesion relaxation of endothelial cells engaged in tubulogenesis, while preserving cell viability.

Additional evidence for the biological relevance of pro-angiogenic activities of the HBD domain of TSP-1 came from studies performed with endothelial colony-forming cells (ECFC), or endothelial progenitor cells, isolated from human cord blood (Dias et al., 2012). Besides stimulating endothelial tubulogenesis of ECFC, as previously observed with adult primary endothelial cells (HUVECs; Nunes et al., 2008), TSP-HepI peptide strongly potentiated FGF-2 angiogenic activity *in vivo*, in the Matrigel plug model.

Adhesion to established endothelial layers is one of the key steps of endothelial progenitor recruitment to ischemic/inflammatory sites. It was shown that the overnight pre-conditioning of ECFC with soluble TSP-HepI (but not with intact TSP-1) significantly increased the adhesion of progenitors to HUVEC monolayers under shear flow (Dias et al., 2012). Interestingly, pre-conditioning with TSP-HepI also resulted in augmented levels of α6 integrin chain on ECFC surfaces. The presence of a neutralizing anti-SDC-4 antibody during the pre-conditioning of ECFC with TSP-HepI inhibited their adhesion to HUVEC monolayers by 84%. While the exact mechanisms for these effects remain to be explored, these data suggested a role of HBD domain in “priming” SDC-4/α6β1 cooperation.

The study of the crystal structure of the HBD (amino acids 1–240) and its complex with a synthetic pentameric heparin (Tan et al., 2008) has shown that, although the positively charged residues of TSP-HepI and TSP-HepII are well separated on the primary sequence of HBD, they congregate to form a patch in the tertiary structure of the domain. Thus, these heparin-binding motifs used for SDC-4 recognition might be accessible in native N-terminal fragments rapidly cleaved *in vitro* and in variable sizes by several proteases relevant to the vascular compartment (Bonnefoy and Legrand, 2000; Morandi, 2009).

## LRP1 as a Receptor for TSP-1

The low density LRP1 is a large cell surface receptor that mediates the endocytosis of a number of different ligands including apolipoprotein E-enriched lipoproteins, protease inhibitor complexes, and matrix proteins including TSP-1 (Lillis et al., 2005). LRP1 is a member of the low-density lipoprotein (LDL) receptor family, which contains seven members that are closely related including the LDL receptor, very low density lipoprotein (VLDL) receptor, apoE receptor2, multiple epidermal growth factor-like domains 7 (MEGF7), glycoprotein 330 (gp330/megalin/LRP2), LRP1, and LRP1B. Similar to the other family members, LRP1 contains several modular structures including cysteine-rich complement-type repeats, EGF repeats, β-propeller domains, a transmembrane domain, and a cytoplasmic domain (Herz et al., 1990). LRP-1 is expressed by many tissues including liver, lung, and brain and in multiple cell types including hepatocytes, fibroblasts, smooth muscle cells, neurons, and macrophages (Moestrup et al., 1992).

Similar to the other family members, LRP1 is an endocytic receptor that at least partly functions to regulate the concentration of extracellular ligand by transporting these ligands through clathrin-coated pits into intracellular vesicles. LRP1 internalizes more than 40 ligands from the pericellular and extracellular environment, including proteinases, ECM proteins, growth factors, and cell surface receptors (Strickland et al., 2002).

Lipoprotein receptor-related protein 1 recognizes a vast number of different ligands and thus mechanisms must be present to inhibit premature association with ligands in the endoplasmic reticulum and allow for proper targeting to the plasma membrane. To facilitate this, the chaperone receptor associated protein (RAP) binds LRP1 with high affinity at multiple sites and antagonizes ligand binding while it is in the endoplasmic reticulum (Herz et al., 1991; Williams et al., 1992).

Lipoprotein receptor-related protein 1 binds TSP-1 at the N-terminal heparin-binding domain, resulting in internalization and degradation in lysosomes (Chen et al., 1996). Through this internalization, TSP-1 can act as a bridging molecule between LRP1 and extracellular ligands and facilitate their clearance. TSP1/LRP-1 binding has been reported to be an important mechanism of clearing matrix metalloproteinases (MMPs) from the extracellular space (Bein and Simons, 2000; Yang et al., 2001). TSP-1 can also associate with cell surface proteoglycans and undergo endocytosis and degradation in an LRP-1-dependent fashion (Godyna et al., 1995; Mikhailenko et al., 1995). LRP-1 and TSP-1 can also act as co-receptors and in the presence of calreticulin can initiate a signaling cascade that results in focal adhesion disassembly (Orr et al., 2003). In the ovary, TSP-1 has been shown to participate in the clearance of VEGF via LRP1, reducing VEGF bioavailability and inhibiting ovarian follicular angiogenesis (Greenaway et al., 2007). TSP-1 also reduces the bioavailability of fibroblast growth factor 2 and hepatocyte growth factor/scatter factor (HGF/SF; Margosio et al., 2003).

## CD36 as a Receptor for TSP-1

CD36 is a transmembrane glycoprotein that is a member of the class B scavenger receptor family. It functions as a long chain fatty acid translocase and a receptor for TSP-1, TSP-2, and collagen (Silverstein and Febbraio, 2009). Through these interactions, CD36 participates in a wide range of physiological processes, including fatty acid metabolism, atherosclerosis, and angiogenesis. CD36 mediates the inhibition of endothelial cell migration and proliferation by TSP-1. Significant quantities of CD36 are found in platelets, macrophages, adipocytes and endothelial cells (Silverstein and Febbraio, 2009). In general, CD36 is expressed on small, but not large, vessel endothelial cells (Luscinskas and Lawler, 1994). Whereas the expression of integrins on tumor vessels was found to vary with the stage of tumor progression in the Rip-Tag model of pancreatic cancer, CD36 is expressed at all stages (Xie et al., 2011). CD36 is a well characterized receptor for TSP-1 in endothelial cells (Jimenez et al., 2000). Engagement of CD36 by TSP-1 activates Fyn, JNK, and p38MAPK to induce apoptosis through activation of caspase-8- and -9-dependent pathways (Jimenez et al., 2000).

The membrane localization of CD36 and its mobility in the plasma membrane has been well studied. CD36 has been reported to be enriched in lipid rafts and tetraspanin-enriched microdomains (Miao et al., 2001; Thorne et al., 2006). The tetraspanins are a family of proteins that contain four transmembrane domains (Hemler, 2014; van Deventer et al., 2020). They undergo homo- and heterotypic association to form distinct regions in the plasma membrane that are enriched in integrins and other membrane proteins. The N- and C-terminals of CD36 contain short intracellular sequences that do not contain consensus sequences for the docking of signal transduction proteins. Thus, CD36 is thought to initiate signal transduction indirectly through complex formation with lipids, integrins and itself. In platelets, the association of CD36 with the Src family kinase Lyn is reportedly mediated by lipids (Thorne et al., 2006). However, the specific Src family kinase that is immunoprecipitated with CD36 depends upon the presence of TSP-1 (Sun et al., 2009). In endothelial cells from wild-type mice, CD36 associates with Fyn and the quantity of Fyn that co-immunoprecipitates with CD36 increases when 3TSR is added. In TSP-1-null endothelial cells, Src replaces Fyn.

In 2006, Daviet et al. (1997) reported that CD36 exists as monomers and dimers in the plasma membrane. Whereas the addition of TSP-1 increases the formation of CD36 dimers in the membrane, TSP-1 did not induce dimerization of a soluble form of CD36. A more recent study reported that over-expression of CD36 in an immortalized endothelial cell line resulted in 40% of the CD36 molecules localizing to nanoclusters that contained as many as 75 CD36 molecules (Githaka et al., 2016). These nanoclusters are enriched in Fyn and F-actin. The size of the clusters and the phosphorylation of Fyn increases after addition of TSP-1 or an anti-CD36 IgM molecule (Githaka et al., 2016). The formation of nanoclusters is likely facilitated by the fact the CD36 mobility in the plasma membrane is restricted by the cytoskeleton (Jaqaman et al., 2011).

**FIGURE 2:**
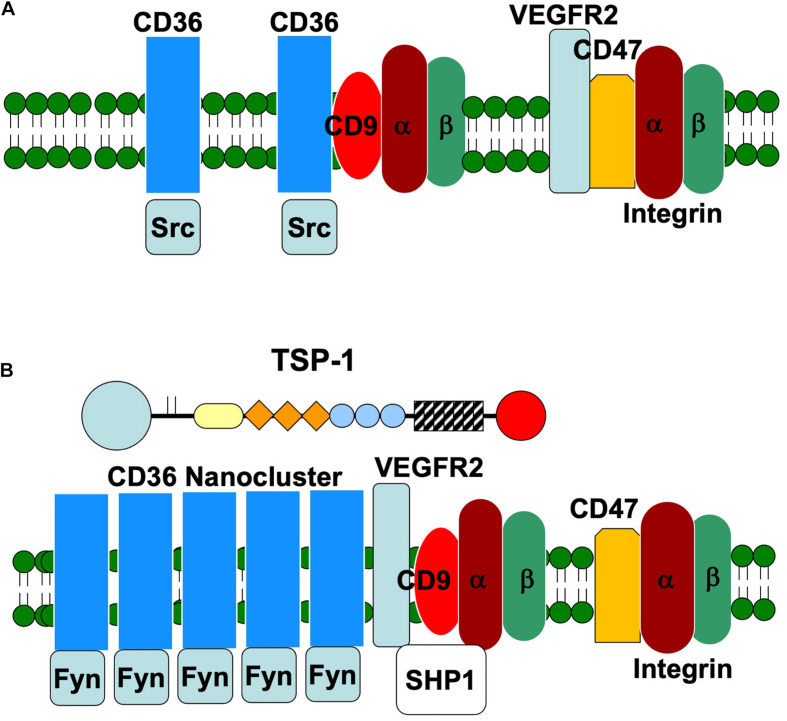
Schematic representation of the organization of endothelial membrane proteins in the absence **(A)** and presence **(B)** of TSP-1. In the absence of TSP-1, Src co-immunoprecipitates with CD36. Fyn is preferentially associated with CD36 in the presence of TSP-1 and the formation of CD36 nanoclusters amplifies Fyn-mediated signal transduction. The presence of TSP-1 also promotes the dissociation of VEGFR2 from CD47 and the association of VEGFR2 with CD36. Integrins and SHP1 also complex with VEGFR2.

The clustering of CD36 promotes signal transduction through recruitment of other membrane proteins, such as integrins, and signaling molecules, such as Fyn and Syk (Figure 2; Kazerounian et al., 2011). Complex formation of CD36 with the tetraspanins CD9, CD81, and CD151 in platelets and endothelial cells has been reported (Miao et al., 2001; Kazerounian et al., 2011). In addition, association with αv, α5, β1, and β2 integrins has been detected (Kazerounian et al., 2011). Since TSP-1 can engage integrins in the absence of CD36 (see below), it may form multivalent complexes with these proteins. In monocytes, the interaction of Syk with CD36 is reportedly mediated by integrins and FcRγ (Heit et al., 2013).

In the context of angiogenesis, the association of CD36 with VEGF receptor 2 (VEGFR2) is a particularly intriguing one because it places receptors involved in pro- and anti-angiogenic signaling in close proximity and raises the possibility that these complexes function as molecular angiogenic switches (Primo et al., 2005). Interestingly, this interaction is markedly reduced in the absence of TSP-1 (Primo et al., 2005; Zhang et al., 2009). VEGFR2 is a key receptor for the stimulation of endothelial cell proliferation and migration by VEGF during physiological and pathological angiogenesis (Simons et al., 2016). The close juxtaposition of a pro-angiogenic signaling receptor with CD36 offers a second pathway through which TSP-1 can antagonize angiogenesis. This pathway involves the recruitment of the phosphatase SHP1 to the CD36/VEGFR2 complex (Chu et al., 2013). SHP1 dephosphorylates VEGFR2 and suppresses the recruitment of pro-angiogenic, downstream signaling proteins. Addition of TSP-1 increases the quantity of SHP1 associated with VEGFR2 in endothelial and ovarian cancer cells (Russell et al., 2015). The inhibition of VEGFR2 phosphorylation by TSP-1 is disrupted when CD36 is mutated so as to lose its ability to interact with β1 integrins (Primo et al., 2005).

Taken together, the data indicate that CD36 forms complexes with multiple other membrane proteins to affect endothelial behavior. Extracellular TSP-1 affects endothelial cell response to stimuli by modulating the composition of these complexes. The complexes that form may be heterogeneous in their composition and size.

## Integrin Binding to TSP-1

The integrins are a family of heterodimeric membrane proteins that function as receptors for extracellular matrix molecules, including collagens, FN, vitronectin, and laminins (Hynes, 2002; Luo et al., 2007). The term integrin was coined to reflect the integral role of these protein complexes as mediators of communication between the extracellular matrix and the cytoskeleton (Tamkun et al., 1986). In addition, a subset of integrins can activate TGFβ (Munger et al., 1999; Mu et al., 2002). An early comparison of platelet GPIIb/GPIIIa and endothelial cell αVβ3 was one of the first indications that integrins are an extensive family of proteins, with various members expressed on virtually all cell types (Charo et al., 1986). Integrins are differentially expressed on large vessel and microvascular endothelial cells, and by various growth factors and cytokines (Luscinskas and Lawler, 1994). As indicated above, integrins are included in tetraspanin-enriched microdomains where they can associate with CD36 and VEGFR-2 in a TSP-1-dependent manner. The αv, α5, β1, and β2 integrins have been reported to be present in these complexes (Hemler, 2014; van Deventer et al., 2020).

A direct RGD-dependent interaction of TSP-1 with αvβ3 has been reported (Lawler et al., 1988; Chen et al., 2000). The RGD sequence of TSP-1 lies within the type 3 repeats, which are a contiguous set of calcium-binding sites. Removal of calcium results in a significant change in the conformation of the TSP-1 molecule and increases exposure of the RGD sequence (Sun et al., 1992). The structure of the type 3 repeats is stabilized by calcium and intrachain disulfide bonds (Tan et al., 2002). Sun et al. (1992) reported that partial reduction of the disulfide bonds in TSP-1 increases the exposure of the RGD sequence. A follow-up study by Hotchkiss et al. (1998) found a disulfide bond isomerase at the plasma membrane of endothelial cells that increased the accessibility of the RGD sequence of TSP-1. Microvascular endothelial cell attachment to and chemotaxis toward TSP-1 are inhibited by antibodies to β3 (Lawler et al., 1988; Taraboletti et al., 1990). TSP-1 may serve as a substrate for endothelial αvβ3 in wounds where high quantities of TSP-1 are deposited by activated platelets.

TSP-1 inhibits the migration of human umbilical vein endothelial cells, which lack CD36, through an integrin β1-dependent mechanism (Short et al., 2005). This pathway for inhibition of migration appears to involve PI3K. Interestingly, an antibody to β1 integrin also inhibits VEGF-induced migration of microvascular endothelial cells that do express CD36 in the presence of an anti-CD36 antibody (Short et al., 2005). These data underscore the close interaction between CD36 and integrins that may arise from their physical proximity in the plasma membrane.

As described above, the NH_2_ domain of TSP-1 promotes angiogenesis through SDC-4 (Ferrari do Outeiro-Bernstein et al., 2002; Dias et al., 2012). This domain has also been reported to enhance angiogenesis through the α9β1 integrin on microvascular endothelial cells through pathways that involve Erk1/2 and paxillin (Staniszewska et al., 2007). The α3β1 and α4β1 integrins have also been reported to bind to the NH_2_ domain of TSP-1 (Chandrasekaran et al., 2000; Calzada et al., 2004). The binding of α4β1 to TSP-1 was observed with venous but not microvascular endothelial cells (Calzada et al., 2004). The interaction of α3β1 and α4β1 integrins with TSP-1 reportedly promotes angiogenesis.

## CD47 as a Receptor for TSP-1

CD47 is a 50 kDa transmembrane receptor, also known as integrin-associated protein (IAP), that was initially identified as a protein lost from red blood cells in patients with Rh-null hemolytic anemia (Miller et al., 1987). CD47 consists of an extracellular N-terminal IgV domain, five transmembrane domains, and a short C-terminal intracellular tail (Mawby et al., 1994). Four alternatively spliced isoforms of CD47 exist, differing in the length of their cytoplasmic tails (Reinhold et al., 1995). CD47 has two main roles: as a ligand for the signal-regulatory protein alpha (SIRPα) and as a receptor for TSP-1. The CD47- SIRPα axis delivers inhibitory signals for phagocytosis and conveys a “don’t eat me” signal that has important functions in hematopoiesis and innate immune surveillance (Tsai et al., 2010; McCracken et al., 2015; Horrigan and Reproducibility Project: Cancer Biology, 2017). Initial studies reported that CD47 is bound by TSP-1 at two peptide motifs on the carboxy-terminal domain (Gao et al., 2017). While partial conservation of some of these residues is found in TSP-2 and -4, CD47 only binds to TSP-1 with high affinity (Isenberg et al., 2009). Some molecules of CD47 contain proteoglycan side chains that are important for high-affinity TSP-1 binding and signaling (Kaur et al., 2011). The interaction with the proteoglycan moieties may be specific to TSP-1.

Thrombospondin 1 binding to CD47 has an inhibitory influence on VEGF signaling. CD47 constitutively associates with VEGFR2 on endothelial (Kaur et al., 2010) and tumor (Russell et al., 2015) cells. Binding of CD47 by TSP-1 inhibits the association between CD47 and VEGFR-2 and disrupts VEGFR2 phosphorylation (Kaur et al., 2010). Ligation by TSP-1 prevents VEGFR2 autophosphorylation, which decreases activation of endothelial nitric oxide synthase (eNOS) associated with Akt phosphorylation. This is consistent with the increased Akt phosphorylation seen in retinal vasculature (Sun et al., 2009) and in ovarian peri-follicular vasculature (Greenaway et al., 2007) of TSP-1 null mice. Similar to the receptor clustering seen with CD36, recent evidence suggests that clustering of CD47 is also required for the high-affinity binding of TSP-1 (Wang et al., 2020). These results suggest that in addition to the amount of CD47 available, the distribution pattern of the receptor is important in regulating TSP-1 signaling.

Activation of CD47 by TSP-1 ultimately inhibits Ca^2+^/calmodulin-mediated activation of eNOS (Bauer et al., 2010), activation of soluble guanylate cyclase (sGC) by nitric oxide (NO; Isenberg et al., 2006), and downstream activation of cGMP-dependent protein kinase (Isenberg et al., 2008b). At physiologic concentrations of TSP-1 (100–200 pM), CD47 is thought to be the dominant receptor for the ligand in inhibiting activation of soluble guanylate cyclase or cGMP-dependent protein kinase (Isenberg et al., 2006, 2008b). Using a systems biology approach, it was determined that enhancing binding of TSP-1 to CD47 decreases the amount of unbound TSP-1 and protects TSP-1 from cleavage, enhancing the anti-angiogenic effect of the ligand (Rohrs et al., 2016).

By disrupting TSP-1/CD47 signaling, angiogenesis can be enhanced, which can have therapeutic benefit. Both TSP-1 and CD47 null mice have decreased necrosis and improved healing in the cutaneous flap model and in full-thickness skin grafts. Skin grafts implanted on wild-type mice fail due to a lack of angiogenesis and tissue perfusion, while those grafted onto TSP-1 or CD47 null mice survive (Isenberg et al., 2008a). Similarly, pancreatic islet grafts have improved survival in TSP-1 null mice, due to increased angiogenesis (Olerud et al., 2008). Using function blocking antibodies to TSP-1 or CD47 has also been shown to reestablish perfusion in ischemic tissues in rodents and pigs (Isenberg et al., 2007, 2008a). In cancer, CD47 null mice had elevated VEGF and VEGFR2 expression and there was increased angiogenesis and accelerated tumor progression in a syngeneic murine model of prostate cancer (Gao et al., 2017).

In addition to its role in angiogenesis, TSP-1/CD47 binding is also involved in regulating tumor immunity. Ligation of CD47 on immune cells inactivates antitumor adaptive immunosurveillance and directly inhibits TCR-mediated T cell activation (Li et al., 2001, 2002). Through CD47, TSP-1 also reduces VEGF-induced immunosuppression in tumors (Kaur et al., 2014), and enhances antitumor immunity by stimulating CD8+cytotoxic T cells (Soto-Pantoja et al., 2014). TSP-1 signaling via CD47 also regulate NK and DC functions that impact adaptive immunity (Weng et al., 2014).

## Conclusion and Future Directions

In this review, we have highlighted the interaction of TSP-1 with endothelial membrane proteins (Figure 1). Through these many interactions, TSP-1 is able to direct the formation of supramolecular complexes that vary in composition over time to modulate the endothelial cell’s response to environmental stimuli (Kazerounian and Lawler, 2018). The data indicate that endothelial cell behavior is determined at the level of the plasma membrane and thus early in the signal generation. The remodeling of membrane protein complexes that form in response to TSP-1 serves to integrate signals from pro- and anti-angiogenic stimuli. In the presence of TSP-1, VEGFR2 dissociates from CD47 and complexes with CD36, which results in enhanced association with integrins and SHP1, leading to dephosphorylation of VEGFR2 and suppression of VEGF signal transduction. TSP-1 also initiates an anti-angiogenic signal through CD36 that leads to decreased endothelial cell migration and apoptosis. This pathway is amplified by the ability of TSP-1 to drive the formation of CD36 nanoclusters, which in turn cluster Fyn. It is important to determine how the concentrations of TSP-1 and VEGF affect the above processes. Does the quantity of the various supramolecular complexes have a linear effect on the net signal, or are there thresholds that must be exceeded in order to initiate response in a stepwise fashion? Can the negative signal elicited by TSP-1 be reversed at some stages and not at others? In addition, our understanding of the role of integrins and their ligands in the way in which endothelial cells respond to TSP-1 is far from complete.

Since tumor growth experiments in TSP-1-null mice indicate that the absence of the intact molecule leads to increased angiogenesis, the pro-angiogenic activity of the NH_2_ domain may be masked or outweighed by the anti-angiogenic activity of the TSRs and the C-terminal domain (Lawler et al., 2001; Xie et al., 2011). In addition, the type 3 repeats have been reported to inhibit angiogenesis by binding to fibroblast growth factor 2 (FGF2; Colombo et al., 2010). Proteolytic cleavage of TSP-1 may produce pro- and anti-angiogenic domains with distinct biological properties. Indeed, the first study to show that TSP-1 is anti-angiogenic detected a proteolytic fragment of TSP-1 in the condition media of p53 deficient fibroblasts from Li-Fraumeni patients (Dameron et al., 1994). This fragment was produced by a proteolytic cleavage between the HBD and the TSRs. We need to better understand the physiological conditions that lead to the release of the HBD from the intact molecule and the disposition of the two or more fragments that are produced. Do the two fragments have different biodistribution and/or stability? Like the intact molecule, the HBD has multiple ligands, including proteoglycans, LPR1, calreticulin and integrins.

## Author Contributions

All authors listed have made a substantial, direct and intellectual contribution to the work, and approved it for publication.

## Conflict of Interest

The authors declare that the research was conducted in the absence of any commercial or financial relationships that could be construed as a potential conflict of interest.
